# Factors related to the success in women's football–a systematic review

**DOI:** 10.3389/fspor.2025.1602457

**Published:** 2025-06-18

**Authors:** Sihang Wang, Miao Shen, Pei Li, Hongyou Liu

**Affiliations:** ^1^ School of Physical Education and Sport Science, South China Normal University, Guangzhou, China; ^2^ School of Physical Education, Guangdong University of Technology, Guangzhou, China; ^3^ National Demonstration Centre for Experimental Sport Science Education, South China Normal University, Guangzhou, China

**Keywords:** determinant, football tradition, match performance, situational factor, women's football

## Abstract

**Introduction:**

Football success is defined as the achievement of a team in the game, which is measured by the combination of winning championship, higher ranking and better performance. This study reviews the factors influencing the football success in women's football from macro (economy, politics, culture), meso (geographical environment, football tradition, talent development, gender equality, league prosperity), and micro (technical/tactical, physical performance, situational factors) perspectives.

**Methods:**

A systematic search was carried out in the Web of Science, Scopus, and Pub Med database, the search strategy included the terms for the population (“women” OR “female”), the sport (“football” OR “soccer”), the variables (determinant', “success”, “ranking”).

**Results:**

A total of 62 studies were included in the analysis. The findings suggest that at the macro dimension, economic development positively correlates with FIFA rankings, and policy support influences football success. Culturally, a culture that emphasizes creativity and strength can facilitate the success in women's football, while a culture of conservatism and humility hinders it. Regarding meso factors, geographical and climatic conditions, football tradition, the quality of talent development, and the degree of gender equality also emerged as important determinants. At the micro dimension, factors such as scoring first, high-intensity running, ball possession, home advantage, and playing weaker teams are closely associated with match success.

**Conclusion:**

These insights offer evidence-based recommendations for policymakers to promote women's football through increased economic investment, infrastructure development, and prioritized support policies, while coaches are encouraged to optimize training processes and incorporate situational factors to enhance team performance.

## 1 Introduction

Football is the most popular sport in the world (1). Although the sport has been historically dominated by men, women's football has undergone significant political, economic, and social changes in the past two decades (2). These changes show that women's football will have great development potential and important commercial value in the future.

Football success refers to the achievement of a team in the sport of football, measured by a combination of factors such as winning championships, achieving high rankings, and demonstrating superior performance on the field (3). Every team aspires to achieve such football success and tries to achieve their goals by all means. Previous studies have examined the determinants of match success from macro (country), meso (football programme), and micro (player) perspectives (4–6). Hoffmann et al. (2002) was the first research to investigate the factors that affect football performance from the perspective of socio-economic, proposing an “inverted U-shaped association” between economy, culture, football population and performance (7). And the subsequent studies implemented political factors, gender equality (8), football traditions and talent development (9). On the other hand, many researchers analyze the match performance of women's football teams, including technical variables (10–12), physical variables (13–15), situational variables (16–20), and link these indicators with the match outcome to explore their internal and external relationships.

However, previous studies have provided an overview of football success from only a single dimension and lacked a multidimensional analysis, especially in the research of women's football (21–23). Therefore, the purpose of this study was to systematically review these articles on the success in women's football and identify the important that contribute to the football performance. This information may provide valuable insights for enhancing team performance in women's football.

## 2 Methods

### 2.1 Search strategy

The systematic review was conducted following the guidelines of the Preferred Reporting Items for Systematic Reviews and Meta-Analyses (PRISMA) statement. The studies related to women's football were collected and summarised by searching Web of Science, Scopus, and Pub Med databases, the deadline for the search was 30th September 2023. The search strategy included the terms for the population (“women” OR “female”), the sport (“football” OR “soccer”), the variables (“determinant” OR “determining”, “success” OR “succeed”, “impact” OR “influence”, “ranking” OR “position”, “economy” OR “economic”, “politics” OR “political”, “culture” OR “cultural”, “climate” OR temperature, “talent development” OR “talent pool”, “football tradition” OR “football legacy”, “gender equality” OR “gender stereotype”, “match performance” OR “sport performance”, “technical”, “tactical” OR “play style”, “physical performance” OR “running performance”, “situational factor” OR “contextual factor”). Each of these keywords was first carried out independently and then combined into a Boolean search using the AND operator. The results of all databases were combined to generate the overall search outcomes. To ensure maximum retrieval of articles, the keywords in all fields were searched, and the necessary information for this study was extracted.

### 2.2 Inclusion and exclusion criteria

The selection of each article was carried out independently by two authors (SW, MS). In case of disagreement, the selection of articles was judged by more experienced experts (HL, PL) until all authors were in agreement. The criteria for inclusion and exclusion were determined using the PICOS method and the details were as follows: (1) the study involved professional female football players/teams; (2) the result was related to the women's football success or match performance; (3) the language of the article was English. Articles were excluded if they had the following. (1) the match sample was non-professional female football; (2) the study was not related to female football success or match performance; (3) it was a conference abstract. Conference abstract papers do not provide enough information to assess the quality and reliability of their methodology, and the comprehensiveness and depth of the research content may not meet the requirements.

### 2.3 Data collection

The following information was systematically extracted from each study: publication year, author affiliations, research dimensions (e.g., macro, meso and micro dimension), investigated variables (e.g., economic variables, technical and physical variables, etc.), and the effects of these variables on match success (e.g., significant positive association, significant negative association, etc.).

### 2.4 Variable selection

The selection of indicators for this analysis was primarily derived from previous studies into factors influencing football performance (7, 8). For instance, economic, political, cultural, climate, football tradition and gender equality indicators were adopted from Hoffmann et al. (2006) (8), while the framework Sports Policy Factors Leading to International Sporting Success (SPLISS) proposed by De Bosscher et al. (2006) provides information for macro and micro dimension examination of athletic achievement determinants (6). Furthermore, according to the reviewed studies, variables related to talent development and league prosperity were incorporated. Performance analysis theory was additionally applied to holistically evaluate physiological, technical, and contextual determinants of success. Measurement indicators of various factors are shown in Table 1. All association classifications used in this review (e.g., positive and negative association) were extracted directly from the interpretations and conclusions reported by the original articles.

**Table 1:** The measuring indicators of influencing factors.

Dimension	Category	Variables
Macro	Economic	Economic indicators (GNP, Gross National Product; GDP, Gross Domestic Product; GDP per capita, Gross Domestic Product per capita etc.) (7, 8)
	Political	Democratic systems (e.g., socialist, capitalist) (3, 28)
	Cultural	Culture characteristics, and Culture oringin (e.g., Latin, Muslim descend, etc.) (8, 25)
Meso	Geographical Environment	Temperature, altitude, humidity, etc. (8, 42, 43)
	Football tradition	History of hosting/participating in international tournaments, men's legacy,etc. (9, 35, 48)
	Talent Development	Training patterns, coach quality, football population, etc. (3, 28)
	Gender Equality	Gender Inequality Index (GII), female labour participation rate, male-female wage ratio, male-female labour force participation rate, male-female school enrolment rate, etc. (36, 37)
	League Prosperity	League rankings, player distribution in elite leagues etc. 51-52)
Micro	Technical performance	Offensive/defensive metrics (shooting, passing, tackles saving, etc.) (15, 67, 70, 78)
	Tactical Performance	Possession-based strategy, direct play, counterattack. (68, 69)
	Physical Performance	Total distance, high-intensity running, running distance with/without ball possession etc. (15, 64, 65)
	Situational Variables	Match location, match status, the quality of opponent, scoreline, etc. (59, 70)

## 3 Results

### 3.1 Studies selection

**Figure 1:**
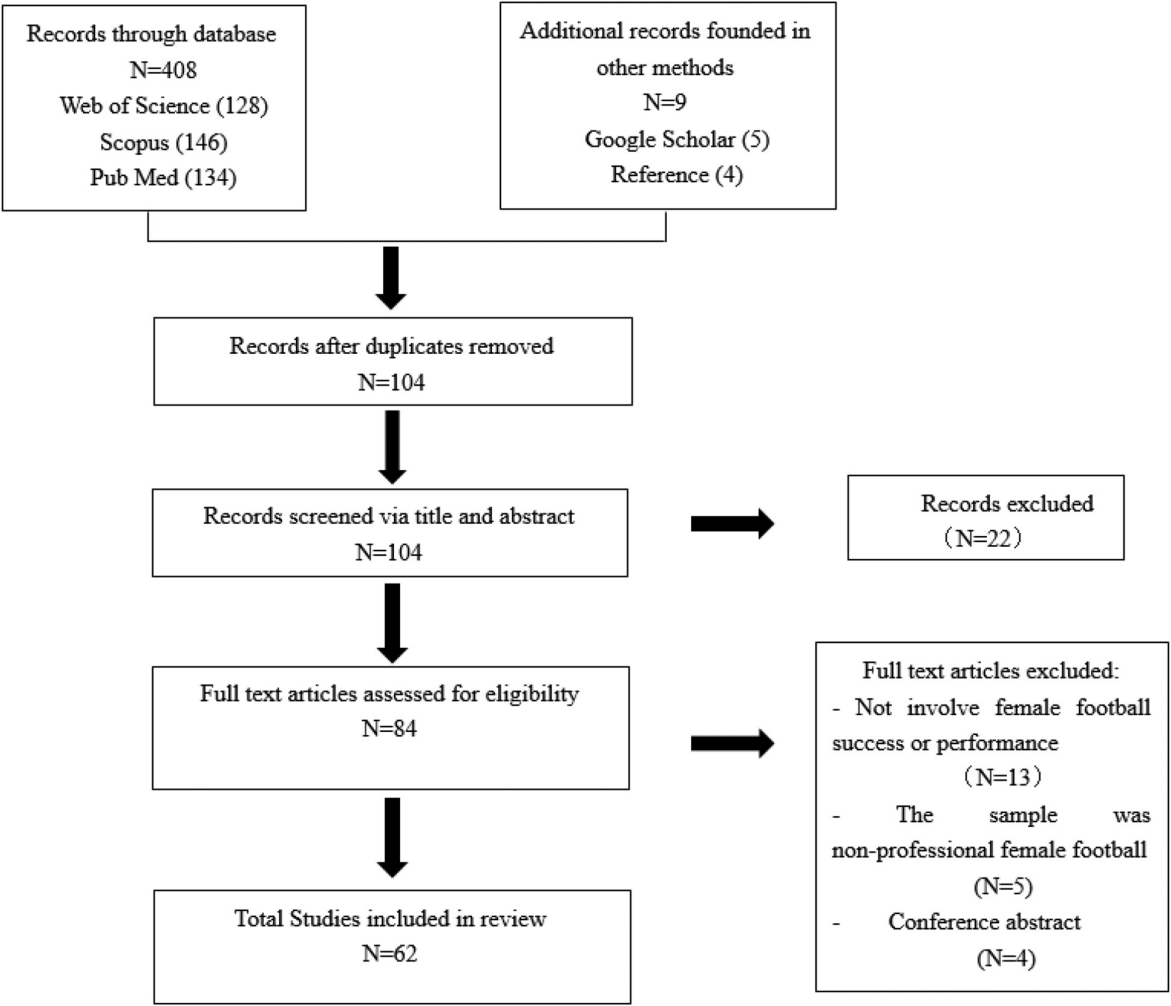
Diagram of article selection process.

An initial search of 417 articles was conducted using keywords. After removing 313 duplicate articles, the remaining articles were screened based on title and abstract. Twenty articles were excluded for they did not fulfil the inclusion criteria. Finally, a total of 62 articles were comprehensively reviewed. The PRISMA flow diagram (see Figure 1) illustrates the process of screening the primary documents.

### 3.2 Study characteristics

A total of 62 studies analyzing factors influencing success and performance in women's football were included (See Table 2). The publication timeline spanned from 2004 to 2023, with an initial low-output phase (2004–2010) averaging 2 articles annually. A substantial increase occurred post-2018, reaching peak productivity during 2020–2023 (*n* = 28, 45.2%), peaking in 2022 with 9 publications. Geographical analysis revealed the United States, Spain, and Australia as the most productive countries (*n* = 9 each, 14.5%), followed by Germany and the United Kingdom (*n* = 6 each, 9.6%). Canada and China contributed 3 studies each (4.8%), while 12 other nations accounted for single publications (1.6% each). The analytical framework comprised three dimensions, macro dimension factors were examined in 20 articles (32.3%), meso dimension in 22 articles (35.5%), and micro dimension in 37 articles (59.7%), 16 articles investigated both macro and meso dimensions. Economic elements dominated macro dimension analyses (*n* = 16, 80%), with 15 articles (93.8%) reporting significant positive correlations. At the meso dimension, football tradition was most prevalent (*n* = 11, 50%), demonstrating at least one consistently positive variable. Micro dimension analyses focused predominantly on technical-tactical performance (*n* = 25, 67.6%), with at least one variable in this factor always positively correlated with match success.

**Table 2:** The study characteristics of articles.

Dimension	Variable	*N* (%)	U	SP	SN	NS
			*N* (%)	*N* (%)	*N* (%)	*N* (%)
Marco dimension (*N* = 20)	Economic factor	16 (80%)		15 (93.8%)		1 (6.3%)
	Political factor	9 (45%)		8 (88.9%)		2 (22.2%)
	Cultural factor	11 (55%)		5 (45.5%)	3* (27.3%)	3* (27.3%)
Meso dimension (*N* = 22)	Geographical environment	7 (31.8%)	3 (42.9%)		2 (28.6%)	2 (28.6%)
	Football tradition	11 (50%)		11* (100%)		1* (9.1%)
	Talent development	10 (45.5%)		10 (100%)		
	Gender equality	10 (45.5%)		10 (100%)		
	League prosperity	6 (27.3%)		6 (100%)		
Micro dimension (*N* = 37)	Technical and tactical performance	25 (67.6%)		25* (100%)	6* (24%)	5* (20%)
	Running performance	15 (40.5%)		11* (73.3%)	2* (13.3%)	13* (86.7%)
	Situational factors	14 (37.8%)		12* (85.7%)	1* (7.1%)	2* (14.3%)

Note: U, inverted U-shaped associtaion; SP, significant positive association; SN, significant negative association;NS, non-significant association; *, study with multiple variables showing different findings. Percentages represent the proportion of articles within each dimension that examined the specific variable. As individual studies may examine multiple variables, totals may exceed 100%.

## 4 Discussion

### 4.1 Macro dimensions

At the macro dimension, this study investigates the impact of economic development levels, political systems, policy support tendency, cultural characteristics, and cultural origins across different countries (regions) on women's football success (see Table 3).

**Table 3:** Macro factors influencing success in women's football.

Author	Macro Indicators				
	Economy	Politics		Culture	
	GDP (per capita)	Policy support	Democratic systems	Culture characteristics	Culture origin
Klein (25)	SP				SN_Muslim_
Caudwell (26)	SP	SP			
Hoffmann et al. (8)	SP	SP	SP		NS _Latin_
Torgler (23)	SP				SP _Latin_
Leeds and Leeds (3)	SP		SP		
Yamamura (27)	SP				
Congdon-Hohman and Matheson (28)	NS		SP		NS _Latin_ SN _Muslim_
Cho (9)	SP				
Manzenreiter et al. (29)		SP		SP	
Jacob (30)	SP		NS		NS _Latin_ SN _Muslim_
Brendtmann (31)	SP				
Klein (32)	SP				
Pfister (33)	SP				
Valenti et al. (24)	SP	SP	NS		
Newman et al. (34)				SP	
Scelles (35)	SP	SP			
Harman (36)	SP		SP		
Özaydın (37)				SP	
Lago et al. (38)	SP				
Narayanan and Pifer (39)				SP	

Note: Latin, the impact of Latin descend; Muslim, the impact of Muslim descend.

#### 4.1.1 Economy

In studies examining economic development levels, many studies use indicators including GDP, per capita GDP and GNP revealing that higher GDP per capita levels are associated with enhanced performance of national women's football teams (23). to quantify the level of economic development (9, 23, 24, 30, 31, 40). Hoffmann et al. (2006) analyzed women's football performance across 88 countries and demonstrated that increased financial investment in women's football positively correlates with improved FIFA rankings (8). Torgler et al. (2008) investigated the relationship between GDP per capita and FIFA Women's World Rankings in 99 countries.

Specifically, nations with higher per capita income exhibit significantly higher rankings for their nation's economic development level positively influences its success in the sport (24). Harman (2022) concurred, noting that this trend is particularly pronounced for countries whose FIFA Women's World Rankings fall outside the top 100 (36). This phenomenon may stem from adequate investment facilitating advancements in football infrastructure, recruitment of professional coaching staff, optimization of talent development systems, and establishment of competitive professional leagues, collectively fostering the growth of women's football and elevating its international competitiveness (8, 25).

#### 4.1.2 Politics

The impact of political factors on football performance is multifaceted. Although football is a sports activity, in reality it is often intertwined with political factors (26). The previous study has indicated that political factors exert a significant impact on the performance of women's football programs. Policy support initiatives, such as China's liberalization policy, have attracted the attention of the education sector and greatly improved the competitiveness of women's football. Another factor is the decision-making of international sports governance, such as expanding the teams participating in the Women's World Cup and adjusting the number of international competitions on different continents, to promote the football participation of emerging countries (35). In addition, the political system has been identified as a key factor in the success of women's football (3, 8, 28). In early research, Hoffmann et al. (2006) analyzed the performance of women's football in 88 countries and found that the socialist system had a significant positive impact on women's football success (8). This finding has been corroborated by subsequent studies (28). Conversely, emerging analyses suggest that socialism negatively affected the development of women's football (36). The reason for this difference may be that under the national background that women's football is generally ignored, socialist countries have achieved short-term first-mover advantage with early financial and policy support, but they have not established a market-oriented professional league model (30). The later capitalist countries have cultivated successful women's professional leagues under market-driven governance and cultivated a sustainable development ecosystem for women's football. This difference accelerates the widening competition gap between the two systems.

#### 4.1.3 Culture

Existing research on the influence of cultural factors on football performance has focused on Latin and Muslim cultures. Hoffmann et al. (2002) collated data from the 2000 Olympic Games and asserted that a 1% increase in the population size of countries of Latin origin would result in an approximately 86-point increase in the FIFA world ranking of men's football (7). However, this cultural advantage is non-significant in the context of women's football, likely due to the constraints imposed by patriarchal norms that suppress feminine athletic expression within Latin cultural matrices (8, 28, 30). This cultural mediation effect becomes more pronounced when examining Muslim contexts. Many studies have indicated that the culture of some Muslim countries has a negative impact on the development of women's football (25, 30, 32). For instance, Congdon-Hohman and Matheson's (2013) study revealed that participation in international women's football is significantly lower in Muslim countries compared to other regions, accounting for a mere 13% of the global participation (28). These observations point to the existence of discernible cultural variations in the realm of women's football. Moreover, women's football teams from Muslim countries generally occupy lower rankings in global standings (30). This phenomenon may be attributed to the presence of structural barriers, which are deeply entrenched in socio-cultural norms and institutional constraints.

In contrast to Asian football culture, Newman et al. (2021) identified Chinese agrarian Confucian traditions as inhibiting creativity and competitive aggressive, conflicting with football's competitive nature (34). Conversely, Japan's bushido ethos fostered a resilient identity in women's football, underpinning their 2011 World Cup victory (41). A contrasting paradigm is evident in the United States, where the success in the 1999 U.S. Women's Soccer Championship paved the way for the continued dominance of women's football by encouraging the active participation of young people through a cultural appreciation of athletic excellence and individual heroism (39).

### 4.2 Meso dimensions

At the meso dimension, the analysis was organized around women's football programme, encompassing geographical environment, historical achievements and male legacy, coach quality, football population, sociocultural acceptance of female athletes' engagement, and professional league prosperity (See in Table 4).

**Table 4:** Meso factors influencing success in women's football.

Author	Meso indicators							
	Geographical environment	Football tradition		Talent development		Gender equality	League prosperity	
	Climate	FIFA ranking	Match experience	Coach quality	Population	Gender equality degree	League ranking	Player distribution
Klein (25)					SP	SP		
Hoffmann et al. (8)	NS	SP	SP		SP	SP		
Torgler (23)	SN	SP	SP	SP	SP			
Leeds and Leeds (3)		SP		SP	SP		SP	
Yamamura (27)		SP					SP	
Zhao et al. (46)		SP						
Congdon-Hohman and Matheson (28)	U	SP		SP	SP	SP		
Cho (9)		NS/SP	NS/SP	SP		SP		
Martínez-Lagunas et al. (14)	U							
Brendtmann et al. (31)	NS				SP	SP		
Klein (32)	U				SP		SP	
Pfister (33)								SP
Valenti et al. (24)				SP	SP	SP		
Pappalardo et al. (48)			SP					
Scelles (35)					SP	SP		
Culvin et al. (49)		SP						
Harman (36)	SN		SP			SP		
Özaydın (37)						SP		
Lago et al. (38)						SP		
Li (50)		SP						
Karlik and Wolden (51)							SP	SP
Thomson et al. (52)								SP

Note: U, inverted U-shaped association.

#### 4.2.1 Geographical environment

Football, as an outdoor sport, is significantly influenced by environmental conditions that directly affect player performance. While previous studies have evaluated geographical factors such as temperature, altitude, and humidity in shaping national team dynamics (42, 43). However, in the context of women's football, many studies have predominantly focused on analyzing temperature's relationship with its performance. Torgler (2008) identified a pronounced negative correlation between elevated temperatures and women's football performance, observing that rising temperatures exacerbate declines in key metrics such as total running distance and high-intensity running output (23). This is consistent with findings in men's football, where heat stress similarly impairs athletic performance due to increased metabolic demands for thermoregulation, diverting critical energy resources from muscular exertion (44, 45). Conversely, cold environments reduce muscle elasticity and joint flexibility, which leads to diminished athletic performance. Prolonged exposure to such conditions adversely affects athletes' capabilities; thus, optimal performance is more achievable in temperate climates. In studies investigating the optimal temperature for football match performance, Congdon-Hohman and Matheson (2013) analyzed matches across over 100 women's national teams and demonstrated that the farther the temperature deviates from 14°C, the greater the decline in performance (28). These findings collectively indicate that temperature exerts a significant influence on athletic performance in football, with a temperature of 14°C identified as the most conducive to optimal outcomes (8, 14, 32).

#### 4.2.2 Football tradition

Football tradition is defined as the historical football achievements in a specific country or region. This encompasses the development level of men's football, and the experience of hosting and participating experiences in international football matches (7). In the study on the association between women's football success and football tradition, Hoffmann et al. (2006) analyzed the tournament outcomes of host nations across 17 World Cups, revealing that 70% of hosts advanced to at least the semi-finals (8). Torgler (2008) investigated women's international football matches in 99 countries and found that the performance would be even better when played as hosts (23). This phenomenon may stem from the logistical and psychological benefits of hosting, which provide coaches and players with critical experience in managing competitive pressure. Additionally, athletes often exhibit heightened national pride in familiar environments, with home crowds further bolstering their self-confidence (9, 36). In addition, previous studies show that there is a correlation between the success in women's football and men's football (27, 28, 46). Cho (2013) compared the FIFA rankings of men's and women's football in different countries and found a significant positive correlation between men's and women's rankings, especially for football in less developed countries (9). Frick and Wicker (2016) analyzed the influence of German men's football on women's football and demonstrated that women's teams adopting tactical frameworks and playing formation from men's football exhibited enhanced competitive success (53). This trend may reflect the delayed emergence of women's football relative to men's, where established men's programs exist, women's teams often adopt their training systems, talent identification frameworks, and tactical approaches (9). Such strategic assimilation of validated methodologies from male football traditions facilitates accelerated performance optimization in women's football, thereby establishing foundational prerequisites for sustained competitive achievement.

#### 4.2.3 Talent development

Talent development constitutes the cornerstone of women's football. Previous studies on measuring the effect of talent development mainly focus on the coach quality, and talent pool in women's football (7). While quantifying coaches' contributions to success remains challenging (47, 54), studies have demonstrated that their expertise and tactical knowledge positively influence performance, as evidenced through structured interviews (55, 56). Valenti et al. (2020) demonstrated a positive impact of higher coaching qualifications on team performance through regression analyses, indicating that more qualified coaches tend to lead to better team results (24). Gómez et al. (2021) further emphasized that tactical innovation, training methodologies, and player management strategies by coaches directly influence team cohesion and competitive outcomes (56).

A critical aspect of talent development lies in expanding the football participation base (57). Empirical research linking national population size to football success has yielded divergent conclusions. Hoffmann et al. (2002) found no significant association between a nation's population and its football success (7), noting that populous countries such as the United States, China, and India are not consistently dominant in men's football. Conversely, Klein (2004) challenged this perspective, identifying a positive relationship between women's football participation rates and competitive performance (25). These findings were corroborated by subsequent studies (3, 25, 28), attributed to the increased likelihood of identifying elite athletes within larger talent pools. Moreover, an active participation atmosphere can increase social interest, broaden grassroots participation and accelerate the development of women's football (23).

#### 4.2.4 Gender equality

While football has historically been stereotyped as a male-dominated sport emphasizing physical duels, aggression, and speed (38), global progress toward gender equality has spurred unprecedented female participation, underscoring the sport's expansive potential (58). This phenomenon raises the key question of whether social acceptance of women's football participation is related to performance gaps in national/regional teams.

However, the degree of gender equality is difficult to quantify precisely, which can only be measured indirectly through some qualitative indicators. Previous studies have quantified the degree of gender equality in countries through gender equality policy (35), GII (37), female labor participation rate (26), male-female wage ratio (8), male-female labor force participation rate^9^, and male-female school enrolment rate (25). The results show that many studies have indicated that countries with higher degrees of gender equality tend to be at the top of women's FIFA ranking (8, 28, 31). Specifically, Özaydın (2022) analyzed European nations, revealing that countries ranked highest in gender equality also featured prominently in women's football standings (37). Torgler (2008) demonstrated that a 10% rise in female labor force participation correlated with a 16.1-point improvement in FIFA rankings (23). Similarly, Congdon-Hohman and Matheson (2013) identified a positive relationship between equitable compensation for female athletes and competitive outcomes (28). This correlation arises because gender-equitable societies provide women athletes enhanced access to professional training infrastructure, equitable remuneration, and stronger institutional support-factors critical to maximizing athletic potential and elevating performance (36).

#### 4.2.5 League prosperity

The enhancement of women's football performance necessitates high-quality competitive platforms for player development, with current research emphasizing the crucial relationship between professional league competitiveness and national team success (27, 32, 33). While the global establishment of professional women's leagues reflects growing recognition (52), significant disparities persist in professionalization levels across national leagues. Empirical evidence demonstrates that elite leagues facilitate technological and tactical transfer through international player recruitment, particularly following the Bosman Law's implementation, with Pfister (2018) confirming that the proportion of players who play in elite leagues is significantly positively correlated with better national team performance. The data from the 2023 Women's World Cup report confirms this phenomenon, showing that the highest representation of 117 participants came from English Women's Super League (EWSL), followed by 74 from Spanish Liga F, where 22 of the 23 members of the Spanish national team competed domestically, underlining the central role of elite leagues in talent development (32, 33). While the United States' historical dominance stems from robust collegiate football systems, the development of its professional leagues has faced challenges such as labor disputes and insufficient financial backing compared to European models, widening the competitive gap. Therefore, to achieve the success in women's football, it is necessary to strengthen the construction of competition and improve the professional league system.

### 4.3 Micro dimensions

From the perspective of the players, the outcome of the match is closely related to their technical, tactical, physical, and psychological performance. Therefore, the analysis of micro-influences on the success in women's football teams must utilize the theory of sports performance analysis, which posits that the side with better sports performance has a higher likelihood of success. At the micro dimension, the performance of women's football teams under the influence of situational factors has been analyzed in terms of technical, tactical, and physical performance, as well as the differences in match outcomes resulting from these indicators. However, most of the studies were conducted independently and only a few were correlated with match outcomes (see Tables 5).

**Table 5:** Micro factors influencing success in women's football.

Author	Micro indicators															
	Technical performance				Tactical performance				Physical performance				Situational factors			
	Ball possession	Shot related	Pass related	Saves	Possession play	Counter attack	Direct play	Total distance	High-intensity	Sprint	Distance with ball	Distance without ball	Match location	Match status	Opponent quality	Scoreline
Gabbett and Mulvey (63)														SP	SP	
Mohr et al. (64)								NS	SP	SP						
Althoff et al.,(65)		SP	SP	SP			SP	NS			SP					
Andersson et al. (66)												NS	SP	SP		
Soroka and Bergier (15)		NS	SP		NS		NS				SP	SN	SP			
Hjelm (67)		SP	SP				SP									
Mara et al. (68)		SP	SP		NS		SP							SP		
Sally and Anderson (69)	NS	SP					SP								SP	
Pollard (70)	NS					SP						NS				SP
Martínez-Lagunas et al. (14)								NS	SP	SP	SP					
Gonzalez-Rodenas et al. (10)	SP	SP	NS		SP	SN										
Hirose et al. (71)								NS	SP	SP	SP	NS				
Hoppe et al. (17)	SP							NS	NS	NS	SP	NS				
Datson et al. (72)								NS	SP	SP	SP	NS				
Ibáñez et al. (59)	SN	SP			SN		SP									SP
Trewin (12)	SP				SP	SN		NS	SP	SP	SP	NS				
Trewin (73)								NS	NS/SP	NS/SP						
Vescovi and Falenchuk (74)													NS			NS
Clarke et al. (75)								NS			SP	NS				
Young et al. (76)	SP	SP														
Casal et al. (77)	SP		SP		NS											
De Jong et al. (78)	SN	SP	SP	SP	SP								SP		SP	
Kubayi (61)	SP	SP	SP		SP	SN	SN						SP	SP	SP	
Maneiro et al. (79)	SP		SP											SP		SP
Scanlan et al. (80)	SP	SP	SP		SP											
Scott et al. (81)								NS	SP	SP						
Wang and Qin (82)	SP	SP	SP		SP											
Scelles (35)													NS			
Maneiro et al. (83)	SP	SP	SP		SP											
Sánchez-Murillo et al. (84)	SN	SP														SP
Dipple et al. (85)		SP	SP													
Mitrotasios et al. (86)	SP	SP	SP				SP									
Iván-Baragaño et al. (87)	SP				SP											
González-Rodenas et al. (88)	SP	SP	SP			SP								SP		
Porras et al. (89)									NS	NS						
Atasever et al. (13)	SP	SP	SP								SP	SN				
Donoghue and Beckley (90)	SP	SP	SP		SP											

#### 4.3.1 Technical and tactical performance

As a technically driven sport in which player skill significantly determines match outcomes (11), women's football exhibits both parallels and divergences from men's football in terms of performance determinants. Ibanez et al. (2018) indicates that scoring the first goal increases the probability of winning by 3%–11%, while the average number of goals in a match is up to 2.53 (59), the trend is consistent with previous studies on men's football (60, 61), although women's matches show greater unpredictability despite higher scoring rates (62). In addition, many studies have confirmed that the variable superiority in possession, passing, and shooting improves the chances of winning. The reason is obvious. Football is a low-scoring sport that typically requires many ball possessions to create shot opportunities and ultimately score goals. However, women's football also has a unique winning factor, De Jong et al. (2020) comparing 700 elite matches identifies intentional assists (defined as deliberate goal-creating passes without deflection) and goalkeeper saves as gender-specific predictors of success (78), which possibly due to reduced defensive intensity and greater team quality differences (59).

At the tactical level, there is no consensus on what kind of tactical play can improve the chances of a women's foootball team winning the game. Althoff et al. (2010) analyzed the 2003 Women's World Cup and found that the elite teams preferred direct attacking play using long passes (65). However, Kubayi & Larkin (2020) argued that women's football is not suitable for long-pass tactical play because it is less effective in women's footall due to the technical limitations of sustaining long passes (61). In contrast, Casal et al. (2020) pushed the idea that more possession play improves the chances of winning matches by analyzing the tactical style of women's football in Spain (77). The reason for this phenomenon may be caused by the applicability of different tactical play styles, the team's characteristics and the differences in the game environment. However, De Jong et al. (2020) argued that the key to winning matches in women's football is the advantage of physical confrontation and that a more aggressive style of play is more likely to win matches (78). This may be since better physicality and aggressive attacking strategies can be effective in limiting opponents and creating more goal-scoring opportunities.

#### 4.3.2 Running performance

Football is a high-intensity sport that demands exceptional running capabilities from players (15). Research investigating the association between running performance and match outcomes in women's football has yielded critical insights. Trewin et al. (2018) analyzed 47 matches from a women's national team and observed that total running distance and low-speed running distance moderately decreased when the team was winning (12). Conversely, Wang and Qin (2020) examined Asian women's football and reported no significant correlation between total running distance and match outcomes (82). This discrepancy may stem from the fact that players predominantly engage in jogging, walking, and jumping during matches, meaning total distance reflects aerobic endurance rather than true running effort. These findings align with the previous studies (19, 66), which similarly concluded that the total distance covered does not predict match success. Instead, high-intensity running variables, such as sprint distance and high-intensity running distance, emerge as determinants (64, 81). Mohr (2008) identified high-intensity running distance as a crucial indicator to differentiate between successful and unsuccessful teams, with the former consistently outperforming the latter (64). Subsequent studies corroborate this, indicating that successful women's teams exhibit superior high-intensity movement profiles, attributable to optimized physical conditioning, tactical discipline, and sustained concentration during play (63, 72, 73). These factors enable greater involvement in attacking and defensive transitions, thereby increasing scoring opportunities.

However, players spend the majority of match time running without ball possession (70). Datson et al. (2017) analyzed international women's matches across the 2011–2012 and 2012–2013 seasons and revealed that successful teams completed more high-speed activities with possession than their counterparts (72). This observation is supported by many studies (12–14, 17, 71, 72, 75), which regard that teams covering greater distances with possession demonstrate superior ball possession percentage, passing accuracy, and attacking efficiency in the opponent's half, thereby minimizing turnover risks (19). Additionally, sustained possession allows teams more time and space to reorganize defensively upon losing the ball, enhancing defensive efficacy (13). Collectively, these advantages underscore the competitive edge conferred by high-intensity running with possession.

#### 4.3.3 Situational variables

The match performance of football teams varies significantly depending on the context of the match (66, 68). Situational variables, such as the quality of the opponent, the location of the match, and the status of the match, have been identified as factors that influence the performance of both the team and the players (68, 61). Previous research on men's football has shown that teams are more likely to win when playing at home (11, 18). However, this advantage is also appeared in women's football (15, 66, 78, 61). Pollard et al. (2014) compared the performances of men's and women's football leagues in 26 countries, it was found that the home advantage of women's football (51%–58.8%) was slightly lower than that of men's football (60%) (70). The reasons for this home advantage may lie in the combination of players' greater familiarity with the playing field, the encouragement of home fans, travelling fatigue, and referee decisions (18).

In addition, many studies have demonstrated that the quality of opponents also significantly affects a team's match performance, with women's football teams scoring fewer goals and having substantially lower chances of winning when playing against stronger teams (15, 65, 78). Iván-Baragaño et al. (2023) examined the effect of opponent's quality on match outcomes in the 2019 Women's World Cup tournament, it was also shown that teams would create more shot and goal scoring opportunities when against weaker opponents, thereby increasing their probability of winning the match (87). The match performance of female players is also affected by different scorelines. As a low-scoring sport, football matches rarely exceed three goals, thus, scoring first exerts a substantial psychological influence, inevitably shaping subsequent performance (74). Teams leading in scorelines have a 70% probability of winning, as demonstrated in prior studies (59, 70, 79). De Jong et al. (2020) analyzed data from 1,390 women's matches and reached an agreement (78). The reason for this may lie in the fact that most teams can adopt a defensive strategy to consolidate their victory when they take the lead.

## 5 Limitations

The limitations of this review should be acknowledged. First, our search strategy was limited to English-language publications, which may have excluded relevant studies published in other languages. Second, we focused exclusively on peer-reviewed journal articles, which may have introduced a selection bias by excluding grey literature such as conference proceedings and institutional reports. Third, the exclusion of unindexed databases and regional repositories may have limited the representation of studies from developing footballing nations. Future systematic reviews would benefit from multilingual search strategies, broader inclusion criteria for evidence sources, and standardised quality assessment tools specifically adapted to sociological sport research.

## 6 Conclusion

The performance of women's football is affected by a combination of factors. At the macro dimension, the development of women's football requires economic, political and cultural support. There is a significant positive correlation between the level of economic development and the FIFA ranking of women's football performance. Policy support contributes to the development of national football, and communist democracy has an important role in promoting the development of women's football in the early stage, but the effect is gradually weakened. Culturally, the culture of heroism and perseverance contributes to improving the performance of women's football. On the contrary, the conservative and humble culture is not conducive. At the meso dimension, successful football traditions, the large size of the female football population, and high degree gender equality are contributed to the women's football performance. At the micro dimension, technical and tactical performance and running performance are important factors affecting the match results. The ball possession, shots, and passes related variables are closely related to match success, but the scoring the first goal has the highest correlation with winning the match. In addition, high-intensity running distance and distance with the ball possession also had a significant effect on the match outcome. Situational factors such as home advantage, playing against weaker teams, and leading the score also contribute to improving match performance. Therefore, this information suggests that policy makers should prioritize increased investment in sports infrastructure and the development of professional women's football leagues, complemented by sustained policy support. Meanwhile, coaches should refine training patterns and incorporate situational factors to strengthen players' technical and tactical abilities, thereby improving team success.

## References

[1] Belozo FL Ferreira EC Lizana CJR Grandim G Machado JC Brenzikofer R The effect of maintaining ball possession on the intensity of games. Motriz: Rev Educ Fis. (2016) 22:54–61. 10.1590/S1980-65742016000100009

[2] Okholm Kryger K Wang A Mehta R Impellizzeri FM Massey A McCall A. Research on women’s football: a scope review. Sci Med Football. (2022) 6(5):549–58. 10.1080/24733938.2020.1868560 36540910

[3] Leeds MA Leeds E. International soccer success and national institutions. J Sports Econ. (2009) 10(4):369–90. 10.1177/1527002508329864

[4] Kite CS Nevill A. The predictors and determinants of inter-seasonal success in a professional soccer team. J Hum Kinet. (2017) 58(1):157–67. 10.1515/hukin-2017-0084 28828086 PMC5548163

[5] Zambom-Ferraresi F Rios V Lera-López F. Determinants of sport performance in European football: what can we learn from the data? Decis Support Syst. (2018) 114:18–28. 10.1016/j.dss.2018.08.006

[6] De Bosscher V De Knop P Van Bottenburg M Shibli S. A conceptual framework for analysing sports policy factors leading to international sporting success. Eur Sport Manag Q. (2006) 6(2):185–215. 10.1080/16184740600955087

[7] Hoffmann R Ging LC Ramasamy B. The socio-economic determinants of international soccer performance. J Appl Econ. (2002) 5(2):253–72. 10.1080/15140326.2002.12040579

[8] Hoffmann R Chew Ging L Matheson V Ramasamy B. International women’s football and gender inequality. Appl Econ Lett. (2006) 13(15):999–1001. 10.1080/13504850500425774

[9] Cho SY. A league of their own: female soccer, male legacy and women’s empowerment. IAI Discuss Pap. (2013) 223:1–28. Göttingen: Ibero-America Institute for Economic Research, Georg-August-Universität Göttingen. 10.2139/ssrn.2228158

[10] Gonzalez-Rodenas J Lopez-Bondia I Calabuig F Pérez-Turpin JA Aranda R. The effects of playing tactics on creating scoring opportunities in random matches from US Major League Soccer. Int J Perform Anal Sport. (2015) 15(3):851–72. 10.1080/24748668.2015.11868836

[11] Pollard R Gómez MA. Components of home advantage in 157 national soccer leagues worldwide. Int J Sport Exerc Psychol. (2014) 12(3):218–33. 10.1080/1612197X.2014.888245

[12] Trewin J Meylan C Varley MC Cronin J. The match-to-match variation of match-running in elite female soccer. J Sci Med Sport. (2018) 21(2):196–201. 10.1016/j.jsams.2017.05.009 28595867

[13] Atasever G Kiyici F. Analysis of match performance indicators of women soccer players in world cups. Online J Recreat Sports. (2023) 12(4):824–8. 10.22282/tojras.1352608

[14] Martínez-Lagunas V Niessen M Hartmann U. Women’s football: player characteristics and demands of the game. J Sport Health Sci. (2014) 3(4):258–72. 10.1016/j.jshs.2014.10.001

[15] Soroka A Bergier J. Actions with the ball that determine the effectiveness of play in women’s football. J Hum Kinet. (2010) 26:97–104. 10.2478/v10078-010-0053-y

[16] Hewitt A Norton K Lyons K. Movement profiles of elite women soccer players during international matches and the effect of opposition’s team ranking. J Sports Sci. (2014) 32(20):1874–80. 10.1080/02640414.2014.898854 24786319

[17] Hoppe M Slomka M Baumgart C Weber H Freiwald J. Match running performance and success across a season in German Bundesliga soccer teams. Int J Sports Med. (2015) 36(6):563–6. 10.1055/s-0034-1398578 25760152

[18] Lago C. The effect of match location, quality of opposition, and match status on possession strategies in professional association football. J Sports Sci. (2009) 27(13):1463–9. 10.1080/02640410903131681 19757296

[19] Rampinini E Impellizzeri FM Castagna C Coutts AJ Wisløff U. Technical performance during soccer matches of the Italian serie A league: effect of fatigue and competitive level. J Sci Med Sport. (2009) 12(1):227–33. 10.1016/j.jsams.2007.10.002 18083631

[20] Redwood-Brown A O’Donoghue P Robinson G Neilson P. The effect of score-line on work-rate in English FA premier league soccer. Int J Perform Anal Sport. (2012) 12(2):258–71. 10.1080/24748668.2012.11868598

[21] Lepschy H Wäsche H Woll A. How to be successful in football: a systematic review. Open Sports Sci J. (2018) 11(1):1–13. 10.2174/1875399X01811010003

[22] Trewin J Meylan C Varley MC Cronin J. The influence of situational and environmental factors on match-running in soccer: a systematic review. Sci Med Football. (2017) 1(2):183–94. 10.1080/24733938.2017.1329589

[23] Torgler B. The determinants of women’s international soccer performances. Int J Sport Manag Mark. (2008) 3(4):305–18. 10.1504/IJSMM.2008.017208

[24] Valenti M Scelles N Morrow S. Elite sport policies and international sporting success: a panel data analysis of European women’s national football team performance. Eur Sport Manag Q. (2020) 20(3):300–20. 10.1080/16184742.2019.1606264

[25] Klein MW. Work and play: international evidence of gender equality in employment and sports. J Sports Econ. (2004) 5(3):227–42. 10.1177/1527002503257836

[26] Caudwell J. Women playing football at clubs in England with socio-political associations. Soccer Soc. (2006) 7(4):423–38. 10.1080/14660970600905711

[27] Yamamura E. Technology transfer and convergence of performance: an economic study of FIFA football ranking. Appl Econ Lett. (2009) 16(3):261–6. 10.1080/13504850601018361

[28] Congdon-Hohman J Matheson VA. International women’s soccer and gender inequality: revisited. In: Robinson L, editor. Handbook on the Economics of Women in Sports. Cheltenham: Edward Elgar Publishing (2013). p. 345–64.

[29] Manzenreiter W. Football in the reconstruction of the gender order in Japan. In: Horne J Manzenreiter W, editors. Football: From England to the World. London: Routledge (2013). p. 78–92.

[30] Jacobs JC. Programme-level determinants of women’s international football performance. Eur Sport Manag Q. (2014) 14(5):521–37. 10.1080/16184742.2014.945189

[31] Brendtmann J Carsten JC Otten S. The effect of gender equality on international soccer performance. Int J Sport Finance. (2016) 11(4):288–309. 10.2139/ssrn.2561223

[32] Klein ML. Women’s football leagues in Europe: organizational and economic perspectives. In: Pfister G Pope S, editors. Female Football Players and Fans: Intruding into a Man’s World. London: Palgrave Macmillan (2018). p. 77–101.

[33] Pfister G. Women, football and European integration: aims, questions, methodological and theoretical approaches. In: Pfister G Pope S, editors. Female Football Players and Fans: Intruding into a Man’s World. London: Palgrave Macmillan (2018). p. 37–54.

[34] Newman JI Xue H Chen R Chen Y Watanabe NM. Football and cultural citizenship in China: a study in three embodiments. Sport Soc. (2021) 24(12):2222–45. 10.1080/17430437.2021.1965125

[35] Scelles N. Policy, political and economic determinants of the evolution of competitive balance in the FIFA women’s football world cups. Int J Sport Policy Politics. (2021) 13(2):281–97. 10.1080/19406940.2021.1898445

[36] Harman J. Gender equality and institutions as the driving force of football performance: women vs men. Athens J Sports. (2022) 9(1):25–36. 10.30958/ajspo.9-1-2

[37] Özaydın S. The impact of gender inequality on women’s team sports-evidence from Europe. Athens J Sports. (2022) 9(2):115–28. 10.30958/ajspo.9-2-4

[38] Lago I Lago-Peñas S Lago-Peñas C. Waiting or acting? The gender gap in international football success. Int Rev Sociol Sport. (2022) 57(7):1139–56. 10.1177/10126902211060727

[39] Narayanan S Pifer ND. A data-driven framing of player and team performance in US women’s soccer. Front Sports Act Living. (2023) 5:1125528. 10.3389/fspor.2023.1125528 36969960 PMC10031044

[40] Andreff W. The correlation between economic underdevelopment and sport. Eur Sport Manag Q. (2001) 1(4):251–79. 10.1080/16184740108721902

[41] Satoshi S. Football, nationalism and celebrity culture: reflections on the impact of different discourses on Japanese identity since the 2002 world cup. In: Manzenreiter W Horne J, editors. Football Goes East. London: Routledge (2004). p. 196–210.

[42] Nassis GP. Effect of altitude on football performance: analysis of the 2010 FIFA world cup data. J Strength Cond Res. (2013) 27(3):703–7. 10.1519/JSC.0b013e31825d999d 22648134

[43] Nassis GP Brito J Dvorak J Chalabi H Racinais S. The association of environmental heat stress with performance: analysis of the 2014 FIFA world cup Brazil. Br J Sports Med. (2015) 49(9):609–13. 10.1136/bjsports-2014-094449 25690408 PMC4413686

[44] Varley MC Gabbett T Aughey RJ. Activity profiles of professional soccer, rugby league and Australian football match play. J Sports Sci. (2014) 32(20):1858–66. 10.1080/02640414.2013.823227 24016304

[45] Mohr M Krustrup P Bangsbo J. Match performance of high-standard soccer players with special reference to development of fatigue. J Sports Sci. (2003) 21(7):519–28. 10.1080/0264041031000071182 12848386

[46] Zhao A Horton P Liu L. Women’s football in the people’s republic of China: retrospect and prospect. Int J Hist Sport. (2012) 29(17):2372–87. 10.1080/09523367.2012.748954

[47] Wicker P Orlowski J Breuer C. Coach migration in German high performance sport. Eur Sport Manag Q. (2018) 18(1):93–111. 10.1080/16184742.2017.1354902

[48] Pappalardo L Rossi A Pontillo G Natilli M Cintia P. Explaining the difference between men’s and women’s football. PLoS One. (2021) 16(8):e0255407. 10.1371/journal.pone.0255407 34347829 PMC8336886

[49] Culvin A Bowes A Carrick S Pope S. The price of success: equal pay and the US women’s national soccer team. Soccer Soc. (2022) 23(8):920–31. 10.1080/14660970.2021.1977280

[50] Li R. The inferior position of female soccer sports: comparison between female soccer and male soccer. J Educ Humanit Soc Sci. (2022) 4:183–7.

[51] Karlik S Wolden M. Women’s collegiate soccer coaching in the United States: exploring barriers and challenges. Soccer Soc. (2023) 24(2):245–57. 10.1080/14660970.2022.2069099

[52] Thomson A Hayes M Hanlon C Toohey K Taylor T. Women’s professional sport leagues: a systematic review and future directions for research. Sport Manag Rev. (2023) 26(1):48–71. 10.1080/14413523.2022.2066391

[53] Frick B Wicker P. The trickle-down effect: how elite sporting success affects amateur participation in German football. Appl Econ Lett. (2016) 23(4):259–63. 10.1080/13504851.2015.1068916

[54] Pieper J Nüesch S Franck E. How performance expectations affect managerial replacement decisions. Schmalenbach Bus Rev. (2014) 66:5–23. 10.1007/BF03396867

[55] Tan TC Zheng J Dickson G. Policy transfer in elite sport development: the case of elite swimming in China. Eur Sport Manag Q. (2019) 19(5):645–65. 10.1080/16184742.2019.1572768

[56] Gómez MA Lago-Peñas C Gómez MT Jimenez S Leicht A S. Impact of elite soccer coaching change on team performance according to coach- and club-related variables. Biol Sport. (2021) 38(4):603–11. 10.5114/biolsport.2021.101600 34937970 PMC8670813

[57] Bernard AB Busse MR. Who wins the Olympic games: economic resources and medal totals. Rev Econ Stat. (2004) 86(1):413–7. 10.1162/003465304774201824

[58] Ahmad A. British football: where are the Muslim female footballers? Exploring the connections between gender, ethnicity and Islam. In: Caudwell J, editor. Women’s Football in the UK. London: Routledge (2013). p. 121–134.

[59] Ibáñez SJ Pérez-Goye JA Courel-Ibáñez J García-Rubio J. The impact of scoring first on match outcome in women’s professional football. Int J Perform Anal Sport. (2018) 18(2):318–26. 10.1080/24748668.2018.1475197

[60] Lago-Peñas C Rey E Lago-Ballesteros J Casáis L Domínguez E. The influence of a congested calendar on physical performance in elite soccer. J Strength Cond Res. (2011) 25(8):2111–7. 10.1519/JSC.0b013e3181eccdd2 21572352

[61] Kubayi A Larkin P. Technical performance of soccer teams according to match outcome at the 2019 FIFA women’s world cup. Int J Perform Anal Sport. (2020) 20(5):908–16. 10.1080/24748668.2020.1809320

[62] Plumley D Mondal S Wilson R Ramchandani G. Rising stars: competitive balance in five Asian football leagues. J Glob Sport Manag. (2023) 8(1):23–42. 10.1080/24704067.2020.1765700

[63] Gabbett TJ Mulvey MJ. Time-motion analysis of small-sided training games and competition in elite women soccer players. J Strength Cond Res. (2008) 22(2):543–52. 10.1519/JSC.0b013e3181635597 18550972

[64] Mohr M Krustrup P Andersson H Kirkendal D Bangsbo J. Match activities of elite women soccer players at different performance levels. J Strength Cond Res. (2008) 22(2):341–9. 10.1519/JSC.0b013e318165fef6 18550946

[65] Althoff K Kroiher J Hennig EM. A soccer game analysis of two world cups: playing behavior between elite female and male soccer players. Footwear Sci. (2010) 2(1):51–6. 10.1080/19424281003685686

[66] Andersson H Ekblom B Krustrup P. Elite football on artificial turf versus natural grass: movement patterns, technical standards, and player impressions. J Sports Sci. (2008) 26(2):113–22. 10.1080/02640410701422076 17852688

[67] Hjelm J. The bad female football player: women’s football in Sweden. Soccer Soc. (2011) 12(2):143–58. 10.1080/14660970.2011.548352

[68] Mara JK Wheeler KW Lyons K. Attacking strategies that lead to goal scoring opportunities in high level women’s football. Int J Sports Sci Coach. (2012) 7(3):565–77. 10.1260/1747-9541.7.3.565

[69] Sally D Anderson C. The Numbers Game: Why Everything you Know About Soccer is Wrong. London: Penguin (2013).

[70] Pollard R Gómez MA. Comparison of home advantage in men’s and women’s football leagues in Europe. Eur J Sport Sci. (2014) 14(Suppl 1):S77–83. 10.1080/17461391.2011.651490 24444247

[71] Hirose N Nakahori C. Age differences in change-of-direction performance and its subelements in female football players. Int J Sports Physiol Perform. (2015) 10(4):440–5. 10.1123/ijspp.2014-0214 25365733

[72] Datson N Drust B Weston M Jarman IH Lisboa PJ Gregson W. Match physical performance of elite female soccer players during international competition. J Strength Cond Res. (2017) 31(9):2379–87. 10.1519/JSC.0000000000001575 27467514

[73] Trewin J Meylan C Varley MC Cronin J Ling D. Effect of match factors on the running performance of elite female soccer players. J Strength Cond Res. (2018) 32(7):2002–9. 10.1519/JSC.0000000000002584 29570576

[74] Vescovi JD Falenchuk O. Contextual factors on physical demands in professional women’s soccer: female athletes in motion study. Eur J Sport Sci. (2019) 19(2):141–6. 10.1080/17461391.2018.1491628 29961405

[75] Clarke AC Couvalias G Kempton T Dascombe B. Comparison of the match running demands of elite and sub-elite women’s Australian football. Sci Med Football. (2019) 3(1):70–6. 10.1080/24733938.2018.1479067

[76] Young CM Luo W Gastin P Tran J Dwyer DB. The relationship between match performance indicators and outcome in Australian football. J Sci Med Sport. (2019) 22(4):467–71. 10.1016/j.jsams.2018.09.235 30352743

[77] Casal CA Maneiro R Ardá A Losada JL. Gender differences in technical-tactical behaviour of LaLiga Spanish football teams. J Hum Sport Exerc. (2020) 16(1):37–52. 10.14198/jhse.2021.161.04

[78] De Jong LMS Gastin PB Angelova M Bruce L Dwyer DB. Technical determinants of success in professional women’s soccer: a wider range of variables reveals new insights. PLoS One. (2020) 15(10):e0240992. 10.1371/journal.pone.0240992 33091064 PMC7580913

[79] Maneiro R Losada JL Casal CA Ardá A. The influence of match status on ball possession in high performance women’s football. Front Psychol. (2020) 11:487. 10.3389/fpsyg.2020.00487 32265794 PMC7104793

[80] Scanlan M Harms C Cochrane Wilkie J Ma’ayah F. The creation of goal scoring opportunities at the 2015 women’s world cup. Int J Sports Sci Coach. (2020) 15(5-6):803–8. 10.1177/1747954120942051

[81] Scott D Haigh J Lovell R. Physical characteristics and match performances in women’s international versus domestic-level football players: a 2-year, league-wide study. Sci Med Football. (2020) 4(3):211–5. 10.1080/24733938.2020.1745265

[82] Wang SH Qin Y. Differences in the match performance of Asian women’s football teams. J Phys Educ Sport. (2020) 20:2230–8. 10.7752/jpes.2020.s3299

[83] Maneiro R Losada JL Casal CA Ardá A. Identification of explanatory variables in possession of the ball in high-performance women’s football. Int J Environ Res Public Health. (2021) 18(11):5922. 10.3390/ijerph18115922 34072971 PMC8198256

[84] Sánchez-Murillo P Antúnez A Rojas-Valverde D Ibáñez SJ. Impact and outcomes of scoring first in professional European female football. Int J Environ Res Public Health. (2021) 18(22):12009. 10.3390/ijerph182212009 34831765 PMC8619132

[85] Dipple JW Bruce L Dwyer DB. Identifying the optimal characteristics of ball possession and movement in elite women’s soccer. Int J Perform Anal Sport. (2022) 22(4):594–603. 10.1080/24748668.2022.2101837

[86] Mitrotasios M Ródenas JG Armatas V Malavés RA. Creating goal scoring opportunities in men and women UEFA champions league soccer matches: tactical similarities and differences. Retos. (2022) 43:154–61. 10.47197/retos.v43i0.88203

[87] Iván-Baragaño I Ardá A Losada J Maneiro R. Influence of quality of opposition in the creation of goal scoring opportunities in women’s football. Apunts Educ Fís Esports. (2023) 154:1–9. 10.5672/apunts.2014-0983.es.(2023/4).154.07

[88] González-Rodenas J Mitrotasios M Armatas V Aranda R. Effects of gender, age and match status on the creation of shooting opportunities during the U17, U20 and senior FIFA world cup: a multilevel analysis. J Hum Sport Exerc. (2023) 18(4):941–53. 10.14198/jhse.2023.184.17

[89] Porras LDR Solano-Mora L Rivas-Borbón M Moreno-Villanueva A Soler-López A Pino-Ortega J Profile of physical demands in female soccer players during competitions: a systematic review. Strength Cond J. (2022) 10:1519. 10.1519/SSC.0000000000000829

[90] O’Donoghue P Beckley S. Possession tactics in the UEFA women’s EURO 2022 soccer tournament. Int J Perform Anal Sport. (2023) 23(1):48–64. 10.1080/24748668.2023.2206273

